# Role of Methyl Prednisolone in Longitudinal Extensive Transverse Myelitis (LETM) in Children

**DOI:** 10.12669/pjms.36.3.1232

**Published:** 2020

**Authors:** Muhammad Azeem Ashfaq, Iram Javed, Muhammad Arshad, Muhammad Rizwan Yaseen

**Affiliations:** 1 Dr. Muhammad Azeem Ashfaq, MBBS, FCPS. Senior Registrar, Department of Paediatric Neurology, The Children’s Hospital & the Institute of Child Health, Lahore, Pakistan; 2 Dr. Iram Javed, MBBS, FCPS. Assistant Professor, Department of Paediatric Neurology, The Children’s Hospital Faisalabad, Pakistan; 3 Dr. Muhammad Arshad, MBBS, FCPS Associate Professor, Sargodha Medical College, Sargodha, Pakistan; 4 Muhammad Rizwan Yaseen, Assistant Professor, Department of Economics, Government College University, Faisalabad, Pakistan

**Keywords:** Longitudinal Extensive transverse myelitis, Efficacy, Methyl prednisolone

## Abstract

**Objective:**

The role of methyl prednisolone in longitudinal extensive transverse myelitis in children is not completely discovered in developing country like Pakistan. So this is the first study which aimed to evaluate the efficacy of methyl prednisolone in longitudinal extensive transverse myelitis in children.

**Methods:**

This is quasi experimental hospital based descriptive prospective study. The data was collected from 34 children admitted in Paediatric Neurology department through Outpatient/emergency department in Children’s Hospital and the Institute of Child Health, Lahore for period of one year from January 2018 to December 2018. The children full filling the inclusion criteria were observed before and after giving injection methyl prednisolone 30mg/kg/dose (maximum dose one Gram irrespective of the body weight) once daily for five days in the form of intravenous infusion.

**Results:**

Complete recovery was seen in 41.2% while 58.8% showed partial recovery. The correlation of response to treatment (recovery) with gender, area of spinal cord involvement, muscle power and autonomic dysfunction is found at significance level of five percent according to Chi square test.

**Conclusion:**

Early consideration and administration of methyl prednisolone in longitudinally extensive transverse myelitis in children can be beneficial and can help to reduce the morbidity.

## INTRODUCTION

Acute transverse myelitis (ATM) presents with acute onset paraplegia or quadriplegia with sensory and autonomic involvement.^1^ Acute transverse myelitis in children being an inflammatory involvement of spinal cord is a rare immune-mediated, demyelinating disorder of central nervous system (CNS).^2^ Its incidence in children is 1.7-2 per million children yearly.^3^

Most of cases are seen in adults while 20% cases are seen below 18 years of age. Mostly cases are idiopathic. Preceding febrile illnesses and vaccination have been reported.^4^ Depending upon the extent of spinal cord involvement, ATM is categorized into short segment and long segment.

Longitudinally extensive transverse myelitis (LETM) is taken to mean myelitis characterized by inflammatory lesion of spinal cord extending over ≥3contiguous spinal cord segments.^5^ LETM occurs in 66%-85% of paediatric ATM.^6^ It appears as hyper intense T2W lesions on magnetic resonance imaging (MRI) of spine. It can be complete or incomplete (partial) dysfunction of the spinal cord.^7^ In acute partial transverse myelitis (APTM), clinical presentation can be mild or asymmetric with involvement of one to two vertebral segments in MRI.

A variety of other acquired inflammatory and demyelinating conditions can be associated with LETM.^8^ Not all LETM are Neuromyelitis Optica Spectrum disorder (NMOSD). It can be rare presentation of Mycobacterium tuberculosis infection as well. Acute partial transverse myelitis and brain MRI abnormalities at initial presentation are significantly predictive of a subsequent diagnosis of multiple sclerosis (MS) in children with ATM.^9^

The role of methyl prednisolone in longitudinal extensive transverse myelitis in children is not completely discovered in developing country like Pakistan. So this is the first study which aims to evaluate the efficacy of methyl prednisolone in longitudinal extensive transverse myelitis. It would give a very useful insight regarding formulation of management protocol of LETM locally as well as internationally.

## METHODS

This is quasi experimental hospital based prospective study and conducted in Department of Paediatric Neurology, Children’s Hospital & Institute of Child Health, Lahore for a period of one year from January 2018 to December 2018 after obtaining approval from the Institutional Review Board (Ref. No.: 30085 dated June 4, 2018). Informed written consent was obtained from parents or guardians. The data was collected by nonprobability consecutive sampling technique of 34 children admitted in the department of Paediatric Neurology through Outpatient/emergency department.

The children of both gender and age ranging from six months to 18 years presenting with acute onset paraplegia or quadriplegia with definite sensory level with or without autonomic involvement of bladder and cardiorespiratory system along with radiologically confirmed findings consistent with longitudinally extensive transverse myelitis with ≥3 contiguous spinal segments involvement were included. While children having spinal cord involvement secondary to other myelocompressive disorders like Pott’s disease, spinal tumors and spinal trauma were excluded. Patients were observed after being scrutinized by TMCWG criteria.^10^ All included patients were subjected to detailed neurological examination, spinal MRI and CSF analysis. Base line Neurological status in terms of motor power, sensory, bladder and bowel disturbance were noted. Additional ancillary investigations were also done in selective patients including serological testing, autoimmune profile like ANA, ENA and aquaporin four antibodies. Data was recorded on predesigned proforma. Injection methyl prednisolone 30mg/kg/dose (maximum dose 1 G irrespective of the body weight) once daily for five days in the form of intravenous infusion was given to all children irrespective of time of presentation. Early administration of methyl prednisolone means that it is given within first week and delayed administration means that it is given after first week of onset of illness. Treatment response is noted at 14 days of treatment in terms of improvement in motor power, sensory or autonomic (bowel, bladder) function. Full recovery means attainment of normal motor power with independent ambulation with minimal or no sensory and sphincter disturbances. While partial recovery means that walking with support with spasticity and some sensory signs and urgency of micturition and constipation. No response means unable to walk, absence of sphincter control and sensory deficit.

Data was analyzed by using SPSS v22. Mean and standard deviation are calculated for quantitative variables like age. Descriptive analysis was done by frequency tables. Frequency and percentage are calculated for qualitative variables like sensory level, autonomic dysfunction, need for mechanical ventilation, findings in MRI spine and CSF analysis. Chi square test is applied to see correlation of different variables with response of treatment.

## RESULTS

Thirty four children were enrolled. There were 20 males (58.8%) and 14 females (41.2%). Female to male ratio 1:1.4. The age of the children ranged from one year to 13 years with mean age of admission 6.08 years (±3.53 SD). Youngest patient was one year old. Out of 34 patients, 25(73.5%) patients had involved lower limbs only and 9 (26.47%) patients had both upper and lower limbs involvement. Among these children, only 1(2.9%) patient required ventilator. Before treatment with steroid therapy, autonomic dysfunction was found in the form of urinary bladder involvement in 12 patients (35.3%) while cardio respiratory dysfunction was found in 7 (20.6%) patient. Six (17.6%) children had thoraco-lumbar while 5(14.7%), 19(55.9%) and 4(1.8%) had cervical, cervico-dorsal and whole spine area respectively involved in MRI spine. One patient 1(2.9%) had cervico-medullary involvement. Majority of patients had cervico-dorsal area involved in MRI spine. Sixteen (47%) children had normal CSF while 18 (53%) children had both pleocytosis and raised protein values. Thirty one 31 (91.2%) and 3 (8.8%) children had normal and raised CRP respectively. Twenty 24 (70.6%) children had history of prior vaccination while 10(29.4%) had no prior history of febrile illness or vaccination (Table-I).

**Table-I:** Clinical, lab profile and spinal MRI findings.

Variable	Categories	Frequency
Gender	Male	20(58.8%)
	Female	14(41.2%)
Prior febrile illness	Yes	24(71%)
	No	10(29%)
Disability at presentation	Involvement of lower limbs only (paraplegia)	25(73.5%)
	Both lower & upper limbs (quadriplegia)	9(26.47%)
Ventilator required	Yes	1(2.9%)
	No	33(97.1%)
CRP	Normal	31(91.2%)
	Raised	3(8.8%)
MRI spine	Cervical	5(14.7%)
	Cervico-dorsal	19(55.9%)
	Thoraco-lumbar	6(17.6%)
	Whole spine	4(1.8%)
MRI brain	Normal	20 (58.8%)
	Abnormal	3(8.8%)
	NA(not available)	11(32.35%)
CSF	Normal	16 (47%)
	Raised Protein	18 (53%)
NMO antibodies	Yes	6 (17.6 %)
	No	12(35.29%)
	NA(not available)	16 (47%)

**Fig.1:**
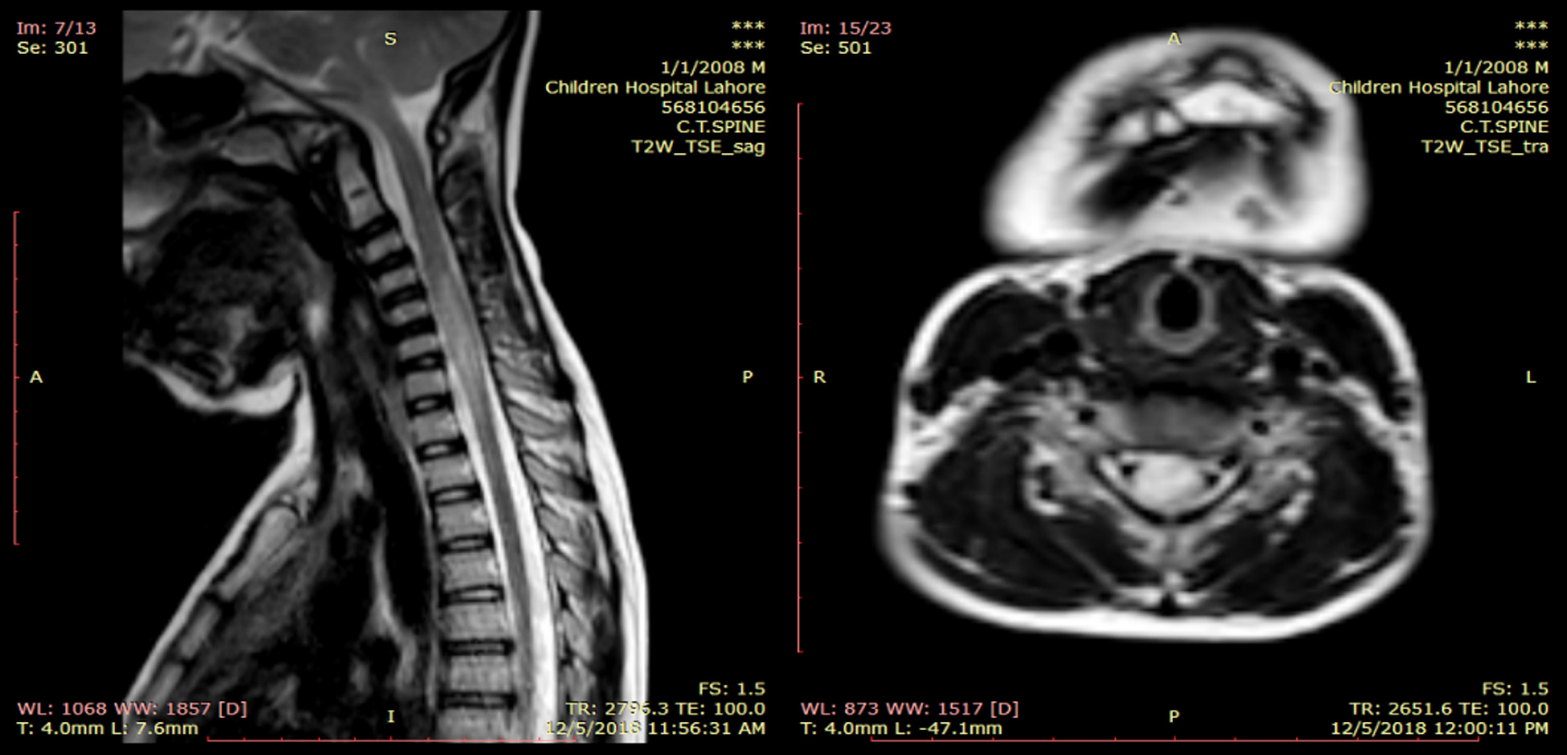
T2 W sagittal and axial MRI through cervical spine. Linear hyperintense area along with swelling within cervical cord (C3-C7) involving upper thoracic cord as well: consistent with long segment Transverse myelitis.

The correlation of response to treatment (recovery) with gender, treatment, area of spine involved, autonomic dysfunction (bladder and cardiorespiratory) is found significant at significance level of five percent (Table-II).

**Table-II:** Correlation between response to treatment and other variables.

	Categories	Response to treatment (Recovery)*		

		Partial n = 20	Complete n=14	P value
Gender	Male	8 (21.1%)	12(35.3%)	0.009
	Female	12 (35.3%)	2 (5.9%)	
Treatment	Early administeration of methyl prednisolone	6(35.3%)	12(21.1%)
	Delayed administeration	14(21.1%)	2(17.6%)
MRI spine area involved	Thoracolumbar	6(17.6%)	0	0.013
	Cervical	3 (8.8%)	2 (5.9%)	
	Cervicodorsal	7(20.6%)	12(35.3%)	
	Whole spine area involved	4(11.8%)	0	
Disability at presentation	Paraplegia	14(41.17%)	11(32.35%)	0.622
	quadriplegia	6(17.64%)	3(8.8%)	
Bladder dysfunction	Yes	4(11.8%)	12(35.3%)	0.000
	No	16 (47.1%)	2(5.9%)	
Autonomic dysfunction of heart	Yes	2(5.9%)	7(20.6%)	0.001
	No	9 (26.5%)	0	
Autonomic dysfunction breathing	Yes	1(2.9%)	6(17.6%)	0.001
	No	9 (26.5%)	0	

* Recovery is taken as categorical variable having two categories i.e. Partial and complete.

After five days of treatment completion, power was assessed on 14^th^ day and had been divided into five grades ranging from 0 (lowest) to five (highest) out of total five. According to results, 0/5 and 1/5 grades of power are observed in 5(14.7%) children. 8(23.5%), 7(20.6%), 6(17.6%) and 3(8.8%) children had 2/5, 3/5, 4/5 and 5/5 grades of power respectively after treatment.

## DISCUSSION

Longitudinally extensive Transverse myelitis (LETM) is of course less common but clinically more important as early diagnosis and adequate and optimum treatment may prevent many patient from significant morbidities.^11^,^12^ LETM as such or as a part of Neuromyelitis Optica Spectrum Disorder (NMOSD) may be triggered by various viral agents besides other infectious causes.^13^ There are no strong controlled trial in children for the most effective treatment of ATM, with one study currently going on by Absoud M et al in UK.^14^ There are very few studies on LETM from developing countries in our region.^15^

In one study^3^, the complete recovery, mild deficit and severe deficit was seen in five children (31.2%), three children and 6 (18.7%) respectively after treatment with methyl prednisolone, but in our study, a complete recovery was also seen in 14 (41.2%) children after treatment. Better outcome was noted in females. In a study conducted in India, Pandey S, et al. concluded that early administration of methyl prednisolone reduces the morbidity in these patients.^16^ Similar observation was made in our study. Delayed administration of methyl prednisolone, significant disability at presentation, and an extensive involvement of spinal cord irrespective of the cause, were associated with severe residual disability.^17^ Hence, the beneficial role of methyl prednisolone is proved in our study contrary to the other study by Kalita and Misra in 2001.^18^

### Limitation of the study

It is that it is a single center study over a short period. We did not use expanded disability status scale Score for measurement of disability as used by study.^19^ NMO antibodies and other serological testing were not performed in all patients due to resource constraints. We did not discuss the etiology of LETM and did not observe long term treatment outcome as it was beyond the scope of our study. We feel that our study has got higher percentage of patients of LETM might be due to the reason that our institute is the biggest referral center across the country. This single center prospective study provides information regarding potential benefit of methyl prednisolone in children of our population.

Despite its limitations, major strength of our study is that it provides an insight to develop treatment protocols in children warranting as first line therapy for early management of this potentially devastating entity in resource limited developing countries like Pakistan, as most of studies are available on western population.^20^,^21^

## CONCLUSION

Early consideration and administration of methyl prednisolone in longitudinally extensive transverse myelitis in children can be beneficial and can help reduce the morbidity.

### Authors’ Contribution:

**MAA:** Conceived the idea and designed framework, Data collection, responsible for accuracy and integrity of work.

**MAA, IJ, MA & MRY:** Did manuscript writing and review.

**IJ & MRY:** Did statistical analysis.
